# A novel allosteric driver mutation of β‐glucuronidase promotes head and neck squamous cell carcinoma progression through STT3B‐mediated PD‐L1 N‐glycosylation

**DOI:** 10.1002/mco2.70062

**Published:** 2025-01-19

**Authors:** Zhonglong Liu, Xiaoyan Meng, Xiao Tang, Jian Zhang, Zhiyuan Zhang, Yue He

**Affiliations:** ^1^ Department of Oral Maxillofacial & Head and Neck Oncology Shanghai Ninth People's Hospital Affiliated to Shanghai Jiao Tong University School of Medicine Shanghai China; ^2^ National Center of Stomatology National Clinical Research Center for Oral Disease Shanghai China; ^3^ Medicinal Bioinformatics Center School of Medicine Shanghai Jiao Tong University Shanghai China

**Keywords:** allosteric driver mutation, glycosylation, head and neck squamous cell carcinoma (HNSCC), PD‐L1, STT3B, β‐glucuronidase (GUSB)

## Abstract

Head and neck squamous cell carcinoma (HNSCC) develops and advances because of the accumulation of somatic mutations located in orthosteric and allosteric areas. However, the biological effects of allosteric driver mutations during tumorigenesis are mostly unknown. Here, we mapped somatic mutations generated from 10 tumor‐normal matched HNSCC samples into allosteric sites to prioritize the mutated allosteric proteins via whole‐exome sequencing and AlloDriver, identifying the specific mutation H351Q in β‐glucuronidase (GUSB), a lysosomal enzyme, as a novel allosteric driver mutation, which considerably encouraged HNSCC progression both in vitro and in vivo. Mechanistically, the allosteric mutation of H351Q remarkably attenuated protein trafficking from the endoplasmic reticulum (ER) to lysosomes, leading to ER retention, in which GUSB‐H351Q facilitated the aberrant N‐glycosylation of PD‐L1 through increasing protein stability and mRNA transcripts of the STT3 oligosaccharyltransferase complex catalytic subunit B, an oligosaccharyltransferase complex. Moreover, GUSB‐H351Q reshaped a more immunosuppressive microenvironment featuring increased infiltration of exhausted CD8^+^ T cells and remodeled tumor metabolism, characterized by increased activity of the purine metabolism pathways and pyruvic acid accumulation. This study provides a mechanism‐driven approach to overcoming HNSCC progression and immune evasion and identifies novel druggable targets based on the presence of GUSB allosteric driver mutation.

## Graphical abstract

Liu et al. identified the specific mutation H351Q in β‐glucuronidase (GUSB) as a novel allosteric driver mutation, which facilitated the aberrant N‐glycosylation of PD‐L1 through increasing protein stability and mRNA transcripts of the STT3 oligosaccharyltransferase complex catalytic subunit B (STT3B). Moreover, GUSB‐H351Q reshaped a more immunosuppressive microenvironment featuring increased infiltration of exhausted CD8^+^ T cells and remodeled tumor metabolism, characterized by increased activity of the purine metabolism pathways and pyruvic acid accumulation.

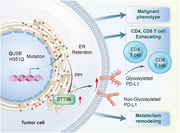

## 1 INTRODUCTION

Head and neck squamous cell carcinoma (HNSCC) is among the most prevalent cancers that pose a significant risk to both human physical and mental health.^1^ Surgery, radiotherapy, chemotherapy, and cetuximab‐based molecularly targeted therapy are currently the primary therapeutic alternatives for HNSCC. The application of immune checkpoint blockades, such as pembrolizumab and nivolumab, has improved the response rate and overall survival (OS) of patients with solid tumors, including HNSCC.^2^, ^3^, ^4^ However, the 5‐year OS of HNSCC patients is reported to be only 50% following this multidisciplinary approach. Identification of more specific approaches for HNSCC patients requires a thorough understanding of the molecular mechanism underlying the development and progression of malignancy.

The development of next‐generation sequencing (NGS) and bioinformatics technology leads to an increasing number of discoveries of tumor driver genes. Four major driver pathways (mitogenic signaling, Notch, the cell cycle, and TP53) and two additional marker genes (FAT1 and CASP8) in HNSCC were identified by Pickering et al. in 2013 via integrated genomic analysis.^5^ Recently, whole‐exome sequencing (WES) has been used to unveil the mutational landscape of HNSCC, identifying two novel driver genes, namely, CHUK and ELAVL1.^6^ More importantly, 58%–80% of HNSCC patients carry at least one mutation in a targetable gene that confers selective progression advantages toward tumor cells, which may indicate that these patients are sensitive and good candidates for current targeted therapies.^5^, ^6^ The discovery of these tumor driver genes allows us to better understand the mechanism of tumor development and progression and provides novel targets for drug development.

Driver mutations typically occur in functional areas, including both allosteric and orthosteric sites. Mutations located in allosteric sites, which are topographically and spatially distinct from the orthosteric site, may also modulate protein activity and serve as tumor driver mutations. Pharmacologically, allosteric ligands are commonly classified as “positive” or “negative” allosteric modulators, with functions that enhance or reduce the biological effect of orthosteric ligands through the initiation of local conformational disturbances and subsequent dysregulation of protein‒protein interactions and communication.^7^ Unlike well‐known orthosteric mutations, the landscape of allosteric driver mutations and their underlying mechanism in cancer progression are less explored. The use of computational methods has made it easier to find allosteric driver mutations. Recently, Zhang et al. developed a platform, AlloDriver, to identify candidate allosteric driver mutations in cancer by analyzing structural and dynamic features to prioritize potentially functional genes/proteins in individual cancers by mapping mutations generated from clinical cancer samples to allosteric/orthosteric sites derived from three‐dimensional protein structures,^8^, ^9^, ^10^, ^11^, ^12^, ^13^, ^14^, ^15^, ^16^, ^17^ which may help to uncover innovative cancer driver genes and the corresponding molecular mechanisms of tumorigenesis through protein alterations, thus identifying new druggable targets.

In this study, we integrated WES and AlloDriver to identify allosteric driver mutations in HNSCC and found that the specific mutation H351Q in β‐glucuronidase (GUSB) was a potential novel molecular mechanism for tumor progression. GUSB is a lysosomal enzyme that is involved in the degradation of glucuronate‐containing glycosaminoglycan (GAG) and is sometimes considered as a housekeeping gene for its relatively stable expression.^18^, ^19^ The overexpression of GUSB has been reported to cause primary resistance to immunotherapy in hepatocellular carcinoma, suggesting a novel strategy for enhancing the efficacy of anti‐PD‐1 therapy.^20^ In vivo GUSB activity can be transformed into urinary signals, which serve as biomarkers for the quantitative monitoring of tumor progression.^21^ However, the functional mutation of GUSB in cancer, especially allosteric sites, has not been elucidated. Through validation of the function of the allosteric site H351Q of GUSB in vitro and in vivo, we report, for the first time, the novel function of the normalization gene GUSB as a pivotal driver mutation in cancer development.

Moreover, we revealed that GUSB‐H351Q is involved in the N‐glycosylation of PD‐L1 through the upregulation of STT3 oligosaccharyltransferase complex catalytic subunit B (STT3B), an oligosaccharyltransferase (OST) complex. Glycosylation is the most diverse protein posttranslational modification and is involved in a complex biological network.^22^ Abnormal glycosylation, especially that of the PD‐L1 checkpoint, has been proven to be a critical factor determining cancer progression. Glycosylation of PD‐L1 promotes protein stability and suppresses T‐cell activity and cytotoxicity, and targeting glycosylated PD‐L1 helps eradicate triple‐negative breast cancer cells.^23^, ^24^ Allosteric mutation of GUSB may be a novel modulator of aberrant PD‐L1 N‐glycosylation in HNSCC, which provides potential methods to inhibit tumor growth and increase immunotherapy efficacy.

Overall, in the present study, we identified a novel allosteric driver mutation site (H351Q) in GUSB that significantly promotes HNSCC progression and modulates the N‐glycosylation of PD‐L1 through the stabilization of the STT3B protein. Moreover, GUSB‐H351Q shapes a more immunosuppressive microenvironment characterized by greater infiltration of exhausted CD8^+^ T cells and a lower distribution of CD4^+^ T cells. This study proposes a mechanism‐driven approach to overcome HNSCC immune evasion and development and provides a theoretical basis for targeted drug research.

## 2 RESULTS

### 2.1 Discovery and validation of the novel driver target GUSB in HNSCC

**FIGURE 1:**
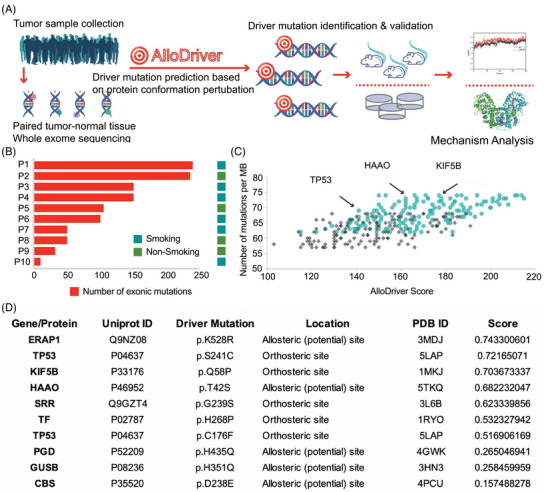
The pipeline and mutation landscape of head and neck squamous cell carcinoma (HNSCC) samples. (A) Schematically description of patient tumor samples based AlloDriver in mutation annotation, mapping, and validation. (B) Exonic mutation landscape of 10 HNSCC samples. (C) The mutational signature analysis across the 10 participants, indicating that AlloDriver can detect driver mutations with low probability and frequency. (D) Top 10 mutational targets and locations were deciphered in the HNSCC cohort.

Ten patients diagnosed with HNSCC were included in this study, and their demographic and clinical information is shown in Table S1. The mean age was 56.5 years (range, 33–74 years) and 40% were female and nonsmokers. To elucidate the mutational landscape for HNSCC, WES was performed on DNA samples from the tumor and paired normal samples with good data quality control with respect to per‐base quality, exome size and coverage depth, and target capture specificity (Figure S1A–C). As shown in Figure 1B, all patients harbored somatic gene alterations, with a median mutation number of 110 (range, 25–240). The mutational signature analysis across the 10 participants revealed that TP53 had the highest frequency of mutations (Figure 1C), which was in accordance with previous literature. Ten potential HNSCC driver targets were then identified via AlloDriver: ERAP1 (p.K528R), TP53 (p.S241C, pC176F), KIF5B (p.Q58P), HAAO (p.T42S), SRR (p.G239S), TF (p.H268P), PGD (p.H435Q), GUSB (p.H351Q), and CBS (p.D238E), most of which have not been previously reported in HNSCC (Figure 1D).

**FIGURE 2:**
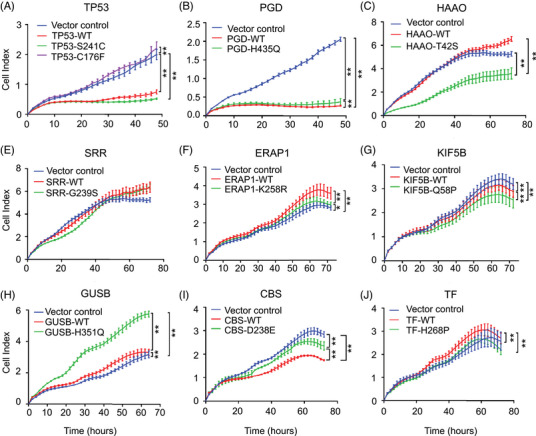
Validation of novel driver mutations in head and neck squamous cell carcinoma (HNSCC). Construction of wild‐type (WT) and mutant (Mt) plasmids and cellular proliferation assay of (A) TP53, (B) PGD, (C) HAAO, (D) SRR, (E) ERAP1, (F) KIF5B, (G) β‐glucuronidase (GUSB), (H) CBS, and (I) TF. Data were represented as mean ± SEM. **p ≤ 0.01; ***p ≤ 0.001; ****p ≤ 0.0001.

To validate the molecular function of these 10 filtered missense mutations in tumor progression, we constructed wild‐type (WT) and mutant (Mt) expression plasmids and then transfected them into the HNSCC cell line HN6 to observe cell viability over time. Significant differences in the proliferation rates were observed between TP53‐WT and the C176F mutant, TAAO‐WT and T42S, GUSB‐WT and H351Q, and CBS‐WT and D238E, among which GUSB‐H351Q dramatically promoted tumor cell growth compared with that of the cells expressing GUSB‐WT and the vector (Figure 2A–I). This growth‐promoting effect of GUSB‐H351Q was further verified in another HNSCC cell line (HN30) (Figure S2). These results indicate that H351Q on GUSB could be an oncogenic driver mutation in HNSCC. GUSB is supposed to play an important role in the progression of HNSCC through perturbation of the allosteric driver mutation H351Q, as suggested by AlloDriver.

### 2.2 Molecular dynamics simulation and intracellular trafficking of the GUSB‐H351Q mutation

**FIGURE 3:**
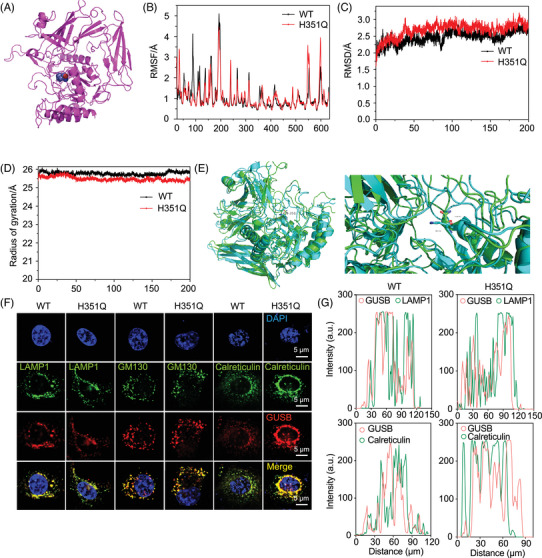
Molecular dynamics simulation and subcellular trafficking analysis of β‐glucuronidase (GUSB). (A) The driver mutation H351Q located at the allosteric site on GUSB (PDB ID: 1BHG). Time evolution of (B) the ligand root means square deviation (RMSD), (C) the root mean square deviation fluctuation (RMSF), and (D) radius of gyration between the GUSB WT and GUSB mut p.H351Q. (E) Structure alignment of GUSB mut p.H351Q. (F) Subcellular distribution of GUSB‐WT/H351Q in lysosomes (LAMP1), the endoplasmic reticulum (calreticulin), and the Golgi (GM130) apparatus under CLSM images. Scale bar: 5 µm. (G) Corresponding quantitative analysis of co‐staining of GUSB (red) with different apparatus markers (green).

Driver mutations occurring at allosteric or orthosteric sites may play determining roles in molecular biological processes. AlloDriver revealed that H351Q in the GUSB protein structure is an allosteric driver mutation, which is characterized by its location at the allosteric site (PDB: 1BHG chain A) in the glycosidase domain of GUSB (Figure 3A). We then aimed to determine whether this allosteric mutation influences the structure and stability of the GUSB protein. Molecular dynamics simulation analysis demonstrated that there was no significant difference in the root mean square deviation (RMSD), root mean square deviation fluctuation (RMSF) curve, or radius of gyration between the GUSB‐WT and H351Q proteins (Figure 3B–D). The similarity of the dynamic fluctuations also indicated that the H351Q mutation caused no damage to the spatial conformation of the protein (Figure 3E). Furthermore, identical trends in the degradation rates of the GUSB‐WT and H351Q proteins were discovered through Western blot analysis of protein samples treated with cycloheximide (CHX), suggesting that the H351Q mutation has little influence on GUSB protein stability (Figure S3).

GUSB is a well‐known lysosomal enzyme that plays a crucial role in the breakdown of glucuronate‐containing GAGs. Hence, we investigated whether the H351Q mutation alters the subcellular distribution of GUSB in lysosomes, the endoplasmic reticulum (ER), and the Golgi apparatus. After WT/H351Q plasmid transfection and expression, HN6 cells were subjected to immunofluorescence staining for GUSB and a lysosomal marker (lysosomal‐associated membrane protein 1 (LAMP1)), an ER marker (calreticulin) and a Golgi marker (Golgi matrix protein [GM130]). Decreased colocalization of GUSB with LAMP1 and increased colocalization of GUSB with calreticulin but not GM130 were observed in H351Q‐transfected cells, indicating that the H351Q mutation has a major effect on GUSB trafficking from the ER to lysosomes (Figure 3F,G). This ER retention may cause functional alterations in the ability of GUSB to participate in ER or lysosomal biological processes. Protein quality control in the ER involves complicated folding and ER‐associated degradation (ERAD). We then investigated whether the ER retention of GUSB‐H351Q leads to protein misfolding and subsequent ER stress. Unfortunately, there was no effect of the GUSB mutation on ER homeostasis, as evidenced by the similar protein expression of Bip, HSP40 and HSP90, which are typical ER molecular chaperones and are the most important indicators of ER stress (Figure S4A). These findings indicated that the mutation of GUSB at the 351 site has no significant influence on protein folding in the ER. Next, we probed the difference in the ERAD level of the GUSB protein. As shown in Figure S4B, the levels of the OS9 and ERLEC1 proteins were greater in the GUSB‐H351Q group than in the WT group, indicating that GUSB‐H351Q attenuated the ERAD pathway, which may lead to ER retention of GUSB.

### 2.3 The GUSB‐H351Q mutation shapes a more progressive phenotype of HNSCC

**FIGURE 4:**
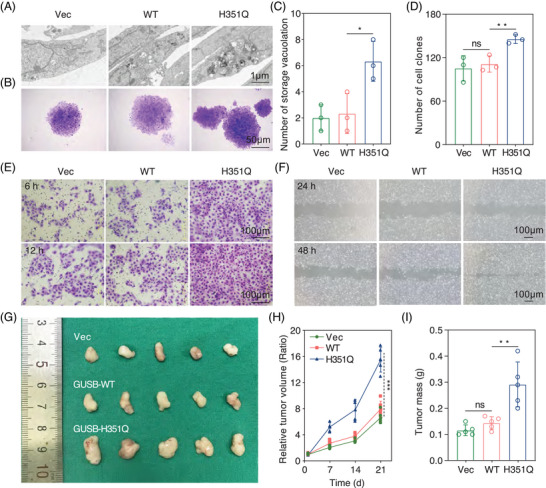
(A) Accumulation of storage vacuolation in GUSB‐Vec/WT/H351Q‐modified HN6 cells under TEM images. Scale bar: 1 µm. (B) Clonal formation assay of HN6 cells overexpressing GUSB‐Vec, WT, and H351Q. Scale bar: 50 µm. (C) Semi‐quantitative analysis of storage vacuolation distribution in tumor cells. (D) Semi‐quantitative analysis of cell clone number among three groups. (E–F) Tumor cell invasion and migration assays of GUSB‐Vec/WT/H351Q‐transfected HN6 cells at designed timepoints. Scale bar: 100 µm. (G) Optical images of dissected HN6 bearing tumors in different groups (*n* = 5). (H) Average tumor growth curves of mice among three groups (*n* = 5). (I) Mean of tumor weights collected from mice at the 21th day (*n* = 5). Data were represented as mean ± SEM. * *p* ≤ 0.05; **p ≤ 0.01; ***p ≤ 0.001. ns, no significance.

The abovementioned findings inspired us to explore whether this abnormal subcellular distribution affects the executive function of GUSB as a lysosomal enzyme. GUSB deficiency is related to intracellular storage vacuolation.^25^ Through transmission electron microscopy, greater accumulation of stored vacuolation was observed in HN6 cells expressing H351Q than in those transfected with the WT plasmid, indicating that the H351Q mutation in GUSB gradually lost its primary function as a lysosomal enzyme (Figure 4A,C). Based on these results, identifying the underlying mechanism by which GUSB‐H351Q performs its biological function in the endoplasmic reticulum is essential.

We subsequently sought to validate the role of the GUSB H351Q mutation in HNSCC progression via colony formation assays. H351Q significantly promoted HN6 cell proliferation, as indicated by the colony formation assays (Figure 4B,D). Tumor cell migration and invasion experiments revealed almost the same trend as that of cell viability (Figure 4E,F). To confirm the functional role of the H351Q mutation of GUSB in remodeling the malignant phenotype, we performed rescue experiments involving the re‐expression of the WT plasmid in H351Q‐transfected HN6 cells and found that GUSB‐WT transfection significantly decreased the cell proliferation induced by the H351Q mutation (Figure S5A). In addition, intervention with GUSB‐WT also counteracted the increased migration and invasion rates of HN6 cells caused by the H351Q mutation (Figure S5B–D).

Furthermore, after stable cell lines were constructed via lentivirus infection (vector, WT or H351Q), we injected genetically modified HN6 cells into the right flanks of BALB/c nude mice to establish a xenograft tumor model and found that compared with the vector, GUSB‐WT had little influence on tumor growth. Nevertheless, H351Q significantly facilitated tumor expansion in vivo, as characterized by the increased tumor volume and weight (Figure 4G–I). Taken together with the in vitro data, these results indicate that the GUSB H351Q mutation remodeled HNSCC cells into a more malignant phenotype and could be a potential oncogenic driver mutation for HNSCC.

The RNA sequencing results revealed significantly differentially expressed genes (DEGs) in the Mut‐OE (GUSB‐H351Q overexpression) group and WT‐OE (GUSB‐WT overexpression) group compared with the Vector group (Figure S6A, B). GUSB expression was upregulated in the Mut‐OE group and the WT‐OE group. The upregulated DEGs included H19, S100A4, C4BPB, S100P, OASL, and CDKN2A in both the Mut‐OE group and the WT‐OE group, while LUM and ALPP were upregulated in the WT‐OE group, and BEGAIN, NPTX1, and PDE3A were upregulated in the Mut‐OE group. The downregulated DEGs included PLAU, MMP14, LAMC2, and CXCL8 (Figure S6C,D). By the Kyoto Encyclopedia of Genes and Genomes (KEGG) pathway enrichment analysis, the upregulated DEGs were enriched in focal adhesion, extracellular matrix (ECM)‐receptor interactions, Rap1 signaling pathways, proteoglycans in cancer, the PI3K‐Akt signaling pathway, and Ras signaling pathway in GUSB‐WT and GUSB‐H351Q groups as compared to the Vector group (Figure S6E,F).

### 2.4 Proteomic identification of STT3B as a core regulator of GUSB‐H351Q

**FIGURE 5:**
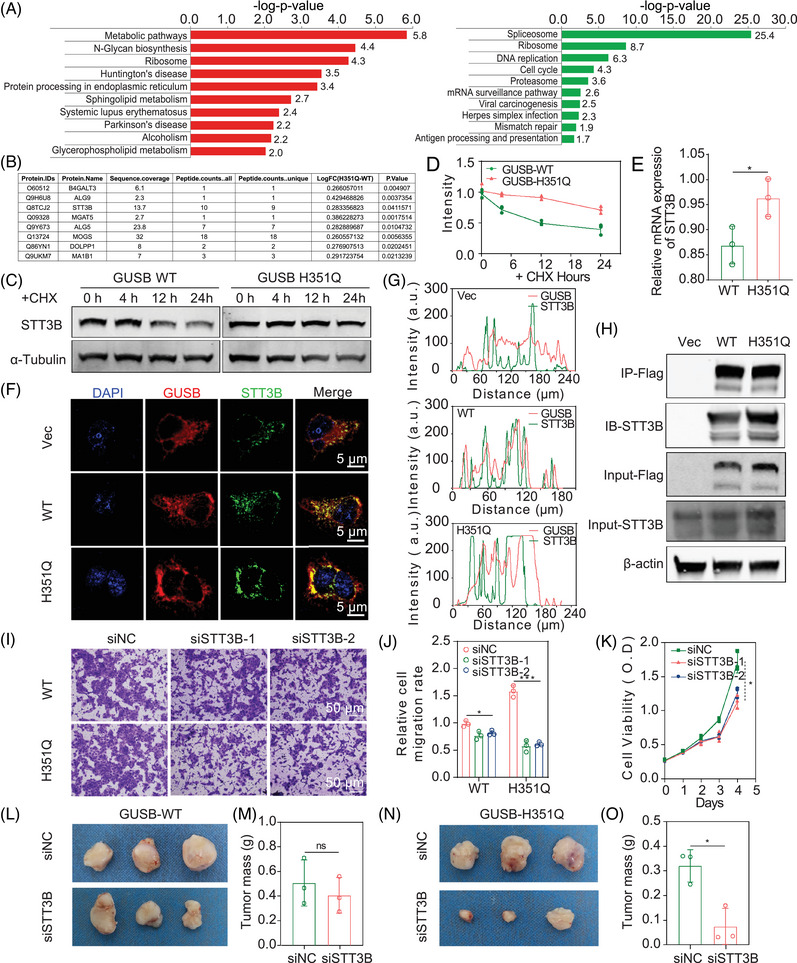
(A) Kyoto Encyclopedia of Genes and Genomes (KEGG) enrichment of differentially expressed proteins (DEPs) following proteomic analysis. (B) Top eight glucosyltransferases participated in N‐glycan synthesis. (C) Protein stability of STT3 oligosaccharyltransferase complex catalytic subunit B (STT3B) between GUSB‐WT and H351Q groups in the presence of cycloheximide (CHX). (D) Time evolution of the STT3B protein turnover rate between two groups. (E) The transcriptome difference of STT3B in GUSB‐WT‐ and H351Q‐transfected HN6 cells. (F) Subcellular co‐distribution of GUSB with STT3B under CLSM images. Scale bar: 5 µm. (G) Corresponding quantitative analysis of co‐staining of GUSB (red) with STT3B (green). (H) Co‐immunoprecipitation (Co‐IP) assay of GUSB with STT3B among three groups following Flag‐tag enrichment. (I) Tumor cell invasion assays of HN6 cells overexpressing GUSB‐WT and H351 followed by STT3B knockdown. Scale bar:50 µm. (J) Semi‐quantitative analysis of invasion capability among four groups. (K) Cell viability assay using CCK‐8 in STT3B‐modified HN6 cells. (L) Optical images of dissected HN6‐WT‐bearing tumors in siNC and si‐STT3B groups (*n* = 3). (M) Average tumor weight acquired from mice at the 21th day (*n* = 3). (N) Optical images of dissected HN6‐H351Q bearing tumors in siNC and si‐STT3B groups (*n* = 3). (O) Average tumor weight acquired from mice at the 21th day (*n* = 3). Data were represented as mean ± SEM. *p ≤ 0.05; ***p ≤ 0.001. ns, no significance.

To delineate the underlying mechanism responsible for GUSB‐H351Q‐mediated protumor efficacy, we performed proteomic analysis to identify the differentially expressed proteins (DEPs) and related pathways. KEGG analysis revealed greater enrichment of metabolic pathways, N‐glycan synthesis and protein processing in the endoplasmic reticulum and lower accumulation of spliceosomes and antigen processing and presentation in GUSB‐H351Q than in GUSB‐WT (Figure 5A). Among these, metabolic shifts or alterations in a more progressive phenotype of cancer have been widely investigated.

Tumor cells commonly present aberrant cell surface glycosylation because of abnormal expression of glycosyltransferases, resulting in changes in typical glycan structures, which may be associated with tumor immune evasion, progression or even metastasis.^26^, ^27^, ^28^ Hence, we focused on the role of H351Q mutation‐mediated abnormal N‐glycan synthesis in HNSCC progression. By screening the DEP list and proteins involved in N‐glycan synthesis, we preliminarily identified several biomarkers, including B4GALT3, ALG9, STT3B, MGAT5, ALG5, MOGS, DOLPP1, and MA1B1, as downstream candidates for GUSB‐H351Q (Figure 5B). Further verification was performed at both the mRNA and protein levels, and STT3B was ultimately identified as a potential mediator of GUSB. Protein expression is associated with the biological processes of biosynthesis and degradation. In the presence of the protein synthesis inhibitor CHX, a slower turnover rate of STT3B was observed in H351Q‐transfected cells than in WT‐transfected cells, indicating that STT3B in GUSB‐WT‐transfected cells was less stable and more susceptible to degradation than that in GUSB‐H351Q‐transfected cells was (Figure 5C,D). Moreover, H351Q induced a slightly higher level of STT3B mRNA transcription than did the WT (Figure 5E). Furthermore, GUSB‐H351Q‐mediated overexpression of STT3B was verified in two other HNSCC cell lines, HN30 and Cal‐27, which presented trends identical to those observed in HN6 cells (Figure S7A). These data suggested that the GUSB‐H351Q‐mediated upregulation of STT3B was mainly ascribed to the increased stability of the STT3B protein and was partially due to the increase in mRNA transcription. To confirm the induction role of GUSB‐H351Q at the intracellular STT3B level, we also carried out rescue experiments by retransfecting GUSB‐H351Q‐expressing cells with GUSB‐WT plasmids and found that overexpression of GUSB‐WT dramatically attenuated STT3B expression at both the transcriptional and protein levels (Figure S7B,C).

STT3B is an ER‐associated N‐glycosyltransferase, and the catalytic subunits of the OST complex participate in the initiation of N‐glycosylation. In addition, the H351Q mutation led to the retention of the GUSB protein in the ER. Hence, we sought to determine whether STT3B is a binding partner for GUSB in the endoplasmic reticulum. At baseline (vector), few colocalizations of GUSB with STT3B were captured in the immunofluorescence images. A remarkably greater amount of fused yellow immunofluorescence was observed in the H351Q‐modified cells than in the WT‐modified cells (Figure 5F,G). To further support this finding, immunoprecipitation (IP) of GUSB following Flag‐tag enrichment revealed increased binding of STT3B to the GUSB protein in the reconstituted GUSB‐H351Q cells (Figure 5H). These data indicate that the protein‒protein interaction between GUSB‐H351Q and STT3B may also be a pivotal mechanism responsible for aberrant N‐glycan synthesis in HNSCC cells. Next, we investigated whether enhanced binding between GUSB‐H351Q and STT3B leads to STT3B stabilization. Protein degradation involves three processes: the ubiquitin‒proteasome system (UPS), the autophagy‒lysosome pathway (ALP) and ER‐associated degradation (ERAD). To explore which pathway STT3B is dependent on for degradation, we pretreated cells with a protein synthesis inhibitor (CHX) and then incubated them with a UPS inhibitor (MG132), an ALP inhibitor (hydroxychloroquine [HCQ]), and an ERAD inhibitor (bortezomib [BTZ]) and found that, in GUSB‐H351Q‐treated cells, STT3B underwent degradation mainly through the ERAD pathway, followed by the autophagy‒lysosome pathway (Figure S7D). Taken together, these findings indicate that the ERAD of GUSB was attenuated, resulting in GUSB‐H351Q ER retention, which augmented the binding activity of GUSB with STT3B and ultimately promoted the stability of STT3B.

Whether the upregulated expression of STT3B is associated with tumor progression is another important issue that needs to be addressed. Transwell assays demonstrated that knockdown of STT3B significantly attenuated GUSB‐H351Q‐promoted cell migration, whereas it had no dramatic effect on GUSB‐WT‐mediated cell mobility (Figure 5I,J). Consistently, the downregulation of STT3B also resulted in a decrease in cell viability (Figure 5K). With respect to the xenograft model, the suppressed expression of STT3B effectively abrogated the pro‐oncogenic effect of GUSB‐H351Q but not that of GUSB‐WT (Figure 5L–O). These data suggest that STT3B is indispensable for the functional execution of GUSB‐H351Q in the tumor microenvironment.

### 2.5 GUSB‐H351Q catalyzes aberrant PD‐L1 N‐glycosylation through STT3B

**FIGURE 6:**
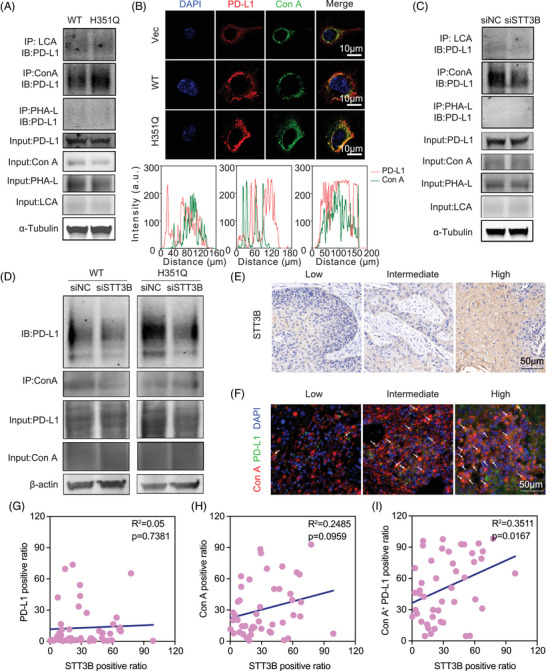
(A) Lectin binding assay (concanavalin A [Con A], lens culinaris agglutinin [LCA], and phytohemagglutinin‐L [PHA‐L]) analyzing the glycosylation status of PD‐L1 protein purified from GUSB‐WT and H351Q cells. (B) Colocalization of PD‐L1 with Con A in HN6 cells expressing GUSB‐Vec, wild type (WT), or H351Q. Scale bar: 10 µm. Under panels show the quantification of the co‐distribution between PD‐L1 and Con A. (C) Lectin blot analysis of the glycosylated PD‐L1 protein following Con A, LCA, and PHA‐L enrichment in HN6 cells transfected with siNC and siSTT3B. (D) Co‐immunoprecipitation (Co‐IP) analysis in detection of the Con A‐modified PD‐L1 in tumor cells co‐transfected with GUSB and STT3B. (E) The representative intensity images for each IHC score of STT3B staining in 46 head and neck squamous cell carcinoma (HNSCC) samples. Scale bar: 50 µm. (F) The representative immunofluorescence images for colocalization of PD‐L1 (green) with Con A (red) in HNSCC samples stratified as low, intermediate, and high. Scale bar: 50 µm. (G–I) The correlation analysis of PD‐L1 positive ration with STT3B positive ratio, Con A positive ration with STT3B positive ratio, and Con A^+^/PD‐L1 positive ratio with STT3B positive ratio.

PD‐L1 glycosylation leads to tumor‐associated immune escape by suppressing T‐cell activity. Targeting glycosylated PD‐L1 could effectively block the PD‐L1/PD‐1 interaction and further promote the PD‐L1 turnover rate and degradation, thus enhancing immune checkpoint therapy.^23^, ^24^ In view of the enriched N‐glycan synthesis pathway in GUSB‐H351Q tumor cells, we probed whether this novel mutation catalyzes the posttranslocational glycosylation of PD‐L1. First, we detected that GUSB‐H351Q had no obvious influence on PD‐L1 expression. Through glycan‐binding lectin enrichment followed by immunoblotting, we identified compelling evidence that the mannose type, but not the core fucose (lens culinaris agglutinin [LCA]) or galactose (phytohemagglutinin‐L [PHA‐L]), was the most crucial N‐glycosylation pattern for PD‐L1, characterized by the high abundance of α‐linked mannose‐modified glycan structures, which could be specifically recognized by the lection concanavalin A (Con A). Surprisingly, GUSB‐H351Q resulted in greater accumulation of PD‐L1 following Con A enrichment than did GUSB‐WT, indicating that the H351Q mutation clearly facilitated the N‐glycosylation of PD‐L1 (Figure 6A). This observation was further verified by the colocalization analysis of Con A lectin with PD‐L1 under immunofluorescence images, which revealed that the level of Con A binding to the PD‐L1 protein was greater in the reconstituted GUSB‐H351 cells (Figure 6B). To confirm the promotive role of GUSB‐H351Q in PD‐L1 N‐glycosylation, we re‐expressed GUSB‐WT plasmids in H351Q‐overexpressing cells and observed that upregulation of GUSB‐WT significantly counteracted H351Q mutation‐mediated aberrant PD‐L1 glycosylation (Figure S8).

As PD‐L1 is widely distributed throughout different cellular compartments, we subsequently investigated PD‐L1 in organelles that underwent GUSB‐H351Q‐mediated N‐glycosylation. Proteins from the nucleus, cytoplasm, and cytomembrane were acquired via cell fractionation and then subjected to co‐IP and lectin blot assays; we found that GUSB‐H351Q mainly dominated the glycosylation of PD‐L1 located at the cell membrane, which may play a pivotal role in signal transduction during the immune response (Figure S9).

STT3B has been reported to participate in stabilization of the immune checkpoint PD‐L1 through the regulation of glycosylation in cancer stem cells.^29^ This finding was also confirmed by our data, as evidenced by a lower accumulation of PD‐L1 following Con A immunoprecipitation in HN6 cells transfected with si‐STT3B (Figure 6C). To further verify this result in HNSCC samples, we scored the STT3B and PD‐L1 glycosylation (a combination of PD‐L1 and Con A) status in a tissue microarray and found that the level of STT3B was positively associated with Con A^+^/PD‐L1 but not with total Con A and PD‐L1 expression (Figure 6E–I, Figure S10). Finally, to determine whether GUSB‐H351Q‐promoted N‐glycosylation of PD‐L1 is predominantly mediated by STT3B, we cotransfected the HNSCC cell line with GUSB and STT3B, and knockdown of STT3B substantially decreased the binding of PD‐L1 with Con A in the GUSB‐H351Q group, suggesting that the GUSB mutation facilitated PD‐L1 glycosylation primarily through STT3B (Figure 6D).

### 2.6 The GUSB‐H351Q mutation induces a more immunosuppressive microenvironment

**FIGURE 7:**
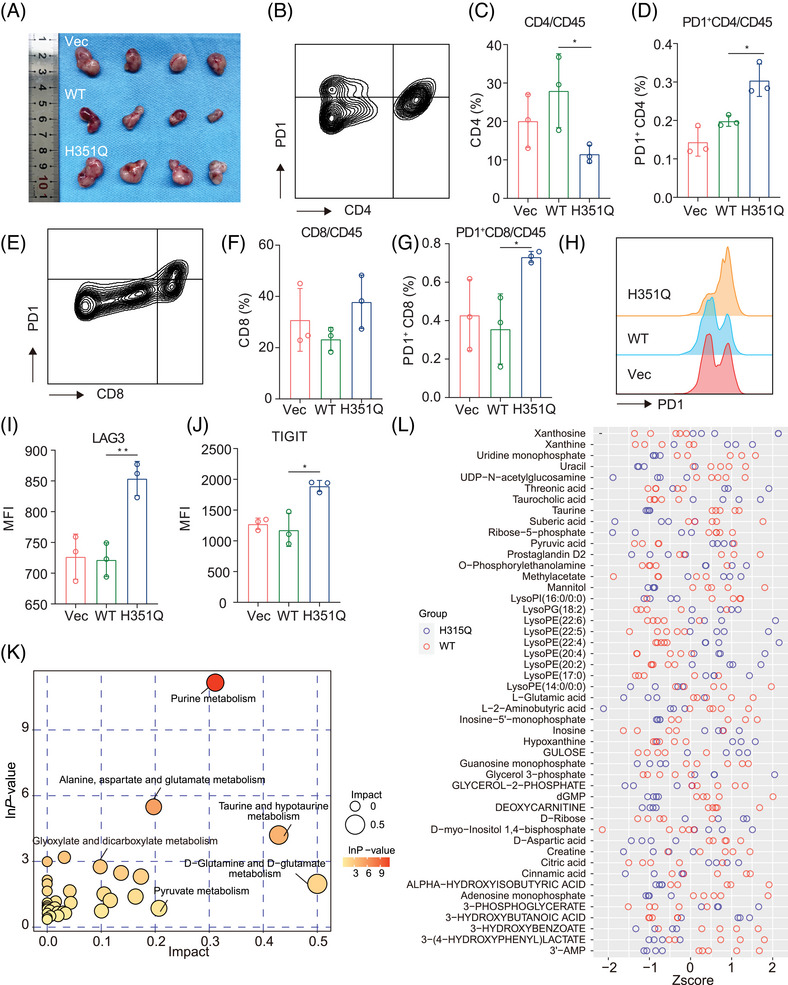
(A) Optical images of dissected SCC7‐Vec/WTH351Q‐bearing tumors in immune complete C3H/He mice (*n* = 4). (B) Fluorescence activated cell sorting (FACS) human papilloma virus analysis of CD4^+^ T cells purified from tumor‐infiltrating lymphocytes (TILs) of SCC7‐bearing tumors. (C) The infiltration percentage of CD4^+^ T cells population in SCC7‐Vec/WTH351Q‐bearing tumors. (D) The infiltration ratio of PD‐1^+^ CD4^+^ T cells population in SCC7‐Vec/WTH351Q‐bearing tumors. (E) FACS analysis of CD8^+^ T cells purified from TILs of SCC7‐bearing tumors. (F) The infiltration percentage of CD8^+^ T‐cells population in SCC7‐Vec/WTH351Q‐bearing tumors. (G) The infiltration ratio of PD‐1^+^ CD8^+^ T cells population in SCC7‐Vec/WTH351Q‐bearing tumors. (H) Flow cytometry measuring of PD‐1 expression on the membrane of CD8^+^ T cells purified from the indicated tumors. (I and J) Flow cytometry measuring of PD‐1 expression on the membrane of CD8^+^ T cells co‐cultured with GUSB‐modified HN6 tumor cells. (K) Metabolic pathway enrichment based on the differential metabolites detected between two groups. (L) *Z*‐score presentation of differential metabolites between GUSB‐WT (*n* = 5) and H351Q (*n* = 4) groups. Data were represented as mean ± SEM. *p ≤ 0.05; **p ≤ 0.01.

GUSB‐H351Q was identified as a previously unrecognized mediator of aberrant PD‐L1 glycosylation characterized by increased expression of complex branched N‐glycans, which are associated with immune evasion and cancer progression. Next, we investigated whether GUSB‐H351Q has a defined role in the immune microenvironment of HNSCC. Through genetic modification of the SCC7 cell line, a xenograft model of HNSCC was successfully established in immunocompetent C3H/He mice. The tumor growth of the GUSB‐H351Q cells was identical to that of the BALB/c nude mice, further confirming the protumoral efficacy of this pivotal mutation (Figure 7A). Moreover, flow cytometry analysis of dissected tumors revealed that GUSB‐H351Q attenuated CD4^+^ T‐cell infiltration and dramatically increased the frequency of PD‐1^+^ CD4^+^ T cells (Figure 7B–D). Although the H351Q mutation had no influence on CD8^+^ T‐cell infiltration, it significantly increased the percentage of PD‐1^+^ CD8^+^ T cells, indicating that H351Q induced a more exhausted phenotype of CD8^+^ T cells (Figure 7E–H). This interesting discovery was further confirmed by the in vitro coculture of tumor cells with activated CD8^+^ T cells, which revealed that the reconstituted H351Q cells promoted the exhausted status of CD8^+^ T cells, as indicated by the increased ratio of LAG3‐ and TIGIT‐positive cells (Figure 7I,J). Taken together, these data reveal that the novel mutation H351Q may suppress the immune response and facilitate immune evasion and tumor progression in HNSCC.

Next, we investigated the metabolic alterations between the GUSB‐WT and H351Q tumor microenvironments. Five tumors from the GUSB‐WT group and four samples from the GUSB‐H351Q group were subjected to liquid chromatography‒mass spectrometry analysis, followed by principal component analysis, partial least squares discriminant analysis (and univariate analysis. Differentially abundant metabolites and their correlations between the two groups are presented as heatmaps of the hierarchical clustering analysis. Further metabolic pathway enrichment revealed that H351Q induced metabolic reprogramming characterized by increased purine metabolism (Figure 7K, Figure S11). Metabolites such as riboflavin, pyruvic acid, threonic acid, lysoPE (22:5), lysoPE (22:4), lysoPE (20:2), xanthine, glycerol 3‐phosphate, and taurocholic acid presented greater accumulation in the GUSB‐H351Q tumors than in the WT tumors, as presented in Figure 7L. Different metabolites may have synergistic or antagonistic effects on the tumor microenvironment. Correlation analysis of various metabolites revealed that pyruvic acid may have synergistic effects with LysoPE (22:5), LysoPE (22:4), and xanthine on tumor progression (Figure S12). Moreover, we investigated effects of metabolic factors on HNSCC progression. Pyruvic acid accumulated in the GUSB‐H351Q tumors compared to the WT tumors (Figure 7L). Therefore, we selected pyruvic acid for further validation experiments. By CCK8 assay, we found that pyruvic acid at 1 M promote cell proliferation while the differences among groups were not drastic (Figure S13A). By transwell assay, we found that pyruvic acid treatment significantly enhanced both migration and invasion capability of HNSCC cells (Figure S13B). Notably, inhibiting PFK (key enzyme of glycolysis and pyruvic acid production) activity significantly reduced their capabilities to the levels of the GUSB‐WT group (GUSB‐WT vs. GUSB‐H351Q+PFKi, ns) (Figure S13C). These results suggested that pyruvic acid may address protumor effects in HNSCC.

## 3 DISCUSSION

Identifying variants that modify protein structure and function is an appealing strategy for deciphering the biological consequences of somatic mutations during tumorigenesis. Allostery, or allosteric regulation, is the biological event in which protein functional activity is altered by the binding of an effector at an allosteric site that is topographically distinct from the orthosteric, active site.^7^, ^30^, ^31^ In the present study, we used AlloDriver to identify a novel allosteric driver mutation, the GUSB H351Q mutation, in HNSCC and validated the function of this allosteric driver mutation both in vitro and in vivo. GUSB encodes a lysosomal enzyme that is involved in the degradation of GAGs.^32^ GUSB gene mutations are reportedly associated with mucopolysaccharidosis (MPS) type VII, an autosomal recessive disease.^33^ To date, the role of GUSB in malignant tumors has not been clearly defined. Seventeen glycolysis‐related genes (GRGs), including GUSB, were found to be dramatically progressive factors in patients with hepatocellular carcinoma. As a member of the GRG signature, GUSB was reported for the first time to be positively associated with poor tumor prognosis.^34^ Inspired by this, a glucose metabolism‐related gene (GMG)‐based model, including LDHA, GUSB, GLB1, GALM, and FBP1, was proposed based on protein‒protein interaction analysis to predict the prognosis of patients with glioma.^35^ As a constituent of the prognostic panel, the mRNA expression of GUSB is positively associated with poor patient survival. At the genomic level, a study of non‐small cell lung cancer in the Mexican population revealed alterations in the mutational profile, including TP53, EGFR, MET, HER2, and GUSB, via targeted NGS. In the present study, we also verified the GUSB mutation in HNSCC and identified a novel mutation, H351Q through AlloDriver that has not yet been reported. However, there are no data concerning whether the GUSB transcript or mutations play a defined role in tumor progression and the underlying mechanism. We first demonstrated that GUSB‐H351Q has a remarkable tumor‐promoting function both in vitro and in vivo and further demonstrated that GUSB‐H351Q increased the N‐glycosylation level of PD‐L1 through STT3B, which indicated that this type of mutation may play an indispensable role in tumor immune evasion.

To date, no investigations have focused on the association of GUSB with glycosylation. Although mainly distributed in lysosomes, GUSB is a member of the glucose metabolic family. The transcription factor THAP1 controls GAG metabolism by enhancing the binding activity of THAP1 with GUSB and regulating GUSB gene expression.^36^ Dectin‐2 recognizes bone marrow‐derived dendritic cells through a mannose‐binding motif. GUSB was identified as a binding partner for dectin‐2, and mutations of the N‐glycosylation sites in GUSB impaired the binding of GUSB to dectin‐2. This information may indirectly suggest that GUSB is correlated with the mannose‐binding motif, which participates in the N‐glycosylation of intracellular proteins.^37^ Consistent with these findings, GUSB promoted PD‐L1 N‐glycosylation, which was characterized by high accumulation of mannose. During protein synthesis, GUSB was trafficked through the ER and Golgi, which was verified by the colocalization of GUSB and ER/Golgi markers under baseline conditions. This subcellular distribution of GUSB may provide an important opportunity for its involvement in protein glycosylation. Another key finding was that, compared with GUSB‐WT, GUSB‐H351Q presented greater ER retention, which may account for the increase in glycosylated PD‐L1 in the mutant cells.

STT3B belongs to the OST family and is involved in protein glycosylation. Epithelial–mesenchymal transition in cancer stem‐like cells induces the N‐glycosyltransferase STT3 through β‐catenin signaling, which enhances PD‐L1 glycosylation and protein stability.^29^ Mammalian OST has two isoforms, the STT3A and STT3B subunits, which catalyze cotranslational and posttranslational N‐glycosylation, respectively.^38^ In other words, the STT3A complex interacts directly with the protein translocation channel to mediate cotranslational glycosylation, whereas the STT3B complex can catalyze posttranslocational glycosylation.^39^ STT3B‐dependent posttranslational N‐glycosylation serves as a triage‐salvage system responsible for the preservation of secretory protein homeostasis.^40^ In the present study, we also demonstrated that STT3B is associated with PD‐L1 glycosylation, which is characterized by the enrichment of the Con A lectin. Most importantly, GUSB‐dependent N‐glycosylation of PD‐L1 occurred mainly through increases in the binding activity and mRNA expression of STT3B.

GUSB has been less frequently reported to participate in the immune response, especially in the tumor microenvironment. In the uveal melanoma cohort, a six‐molecule signature (ARPC1B, BTBD6, GUSB, KRTCAP2, RHBDD3, and SLC39A4), which was associated with glycolysis and the immune response, was established and used for survival prediction and risk stratification of these patients.^41^ Similarly, a glucose metabolism‐related gene‐based model (LDHA, GUSB, GLB1, GALM, and FBP1) was constructed via a protein‒protein interaction network and was found to be positively related to a worse prognosis and greater M2 macrophage infiltration.^35^ More recently, to elucidate the underlying mechanism of resistance to anti‐PD‐1 therapy, 10 patients with hepatocellular carcinoma (HCC) who were treated with anti‐PD‐1 therapy were divided into responsive and nonresponsive tumor patients, and the results revealed that GUSB amplification in nonresponding tumor patients was markedly greater than that in responding tumor patients. Subsequent in vitro assays revealed that GUSB facilitated proliferation and downregulated PD‐L1 expression, which led to primary resistance to anti‐PD‐1 treatment in HCC.^20^ These data demonstrated that GUSB is associated with the immune response, immune cell infiltration, and anti‐PD‐1 treatment. Based on our data, the GUSB H351Q mutation had no effect on PD‐L1 expression but significantly promoted N‐glycosylation of PD‐L1 and created a more immunosuppressive microenvironment characterized by a greater proportion of exhausted CD8^+^ T cells.

## 4 CONCLUSIONS AND LIMITATIONS

**FIGURE 8:**
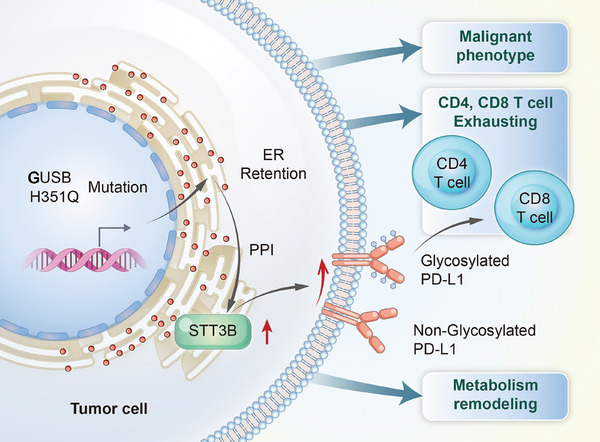
Molecular mechanism of GUSB‐H351Q‐mediated PD‐L1 N‐glycosylation.

In conclusion, the results of the present study revealed a novel allosteric mutation of H351Q in the GUSB protein, which facilitated HNSCC progression characterized by increased proliferation, invasion, and mobility. This protumor effect was attributed to the ER retention of the GUSB‐H351Q protein and subsequent catalysis of PD‐L1 glycan synthesis and aberrant N‐glycosylation, which further increased the percentage of exhausted CD8^+^ T cells and excluded the infiltration of CD4^+^ T cells, creating an immunosuppressive microenvironment (Figure 8). These findings may contribute to the development of HNSCC targeted therapy and even its combination with immune therapy.

However, several limitations exist. First, we focused on the oral squamous cell carcinoma, which could not fully represent HNSCC and constricted the promotion of the results in this study. Second, we did not find specific mutation associated with human papilloma virus (HPV) infections or other clinical risky factors, which may be due to the small sample size or relatively low sequencing depth. Then, we used exogenous GUSB‐H351Q to investigate the impacts of GUSB‐H351Q on HNSCC progression. Moreover, it is difficult to acquire HNSCC samples bearing intrinsic H351Q mutations in GUSB, which impedes the establishment of a patient‐derived tumor xenograft model; thus, the in vivo validation of GUSB‐H351Q‐mediated tumor development and immune evasion is lacking. In addition, the underlying mechanism by which GUSB‐H351Q regulates metabolic remodeling has not been fully elucidated, which will be the focus of our group's future research. Finally, although the pivotal role of the allosteric mutation of GUSB‐H351Q in HNSCC progression has been revealed, the method of the clinical transformation of GUSB‐based targeted therapy is still unclear.

## 5 MATERIALS AND METHODS

### 5.1 Sample collection and whole‐exome sequencing

Tumor and adjacent normal tissues were collected from 10 patients diagnosed with HNSCC at Shanghai Ninth People's Hospital. DNA was extracted from fresh tumor tissues and then subjected to integrity and quality assessments. Genomic profiling was performed through WES on the NextSeq500 platform (Illumina). The average coverage depth after the reads were deduplicated was 30× for the tumor and normal samples.

### 5.2 AlloDriver‐based driver mutation prediction

AlloDriver served as a computational workflow designed to identify therapeutic targets in cancer samples by evaluating how mutations at allosteric (and orthosteric) sites affect protein functions during the proliferation and development of specific cancers, which was followed by an analysis of the anticipated driver mutations within current clinical samples.^10^, ^11^

The schematic description of AlloDriver can be found in Figure 1A. First, we submitted cancer samples to AlloDriver, and missense mutations were detected and mapped to 3D structures of 1650 human proteins originating from the Research Collaboratory for Structural Bioinformatics (RCSB) Protein Data Bank (https://www.rcsb.org/). Mutations occurring at allosteric or orthosteric sites were further examined for driver estimation by structural and dynamic features. Potential driver proteins in query samples as targets were prioritized on the basis of an evaluation of mutations by the AlloDriver score. In addition, for each query sample, the profiling of predicted driver mutations in the human structural proteome was analyzed.

### 5.3 Molecular dynamics simulation assay

To evaluate the structural differences between GUSB‐WT and GUSB‐H351Q, MD simulation analysis was performed via GROMACS 4.5.4 software on a Linux operating system.^42^, ^43^ The WT and mutant complexes were independently simulated for 10, 50, and 200 ns. The ammoniated lignin, which had been energy‐minimized, underwent further annealing for equilibration in a constant‐pressure, constant‐temperature (NPT) Phosphate‐Buffered Saline ensemble at 240°C for 30 ns. We subsequently conducted molecular dynamics (MD) simulations at 30°C intervals ranging from 240°C to −30°C using the final equilibrium structure achieved at the higher temperature as the starting point for the next temperature. For each target temperature, a 100 ns MD simulation was performed, and during the last 50 ns, simulation data were collected every 50 fs for the analysis of the static and dynamic properties of the molecular systems. Using Xmgrace (http: //www. weizmann. ac. il/Grace/) and UCSF Chimera 1.10, the RMSD, RMSF, and radius of gyration analyses were performed.

### 5.4 Animal procedures

Athymic nude mice (BALB/c nu/nu) aged 4–5 weeks were acquired from Shanghai Jihui Laboratory Animal Care Co., Ltd., and housed in sterile cages under laminar airflow hoods in a specific pathogen‐free room at 22°C–25°C with a 12‐h light and 12‐h dark schedule and were fed autoclaved chow and water ad libitum. Our institution approved all the tests, which were carried out in accordance with Shanghai Ninth people's Hospital Guidelines for Animal Care. HN6 cells (3 × 10^6^) were harvested and resuspended in Hank's balanced salt solution and then injected subcutaneously into the right flanks of BALB/c nude mice to establish a xenograft model for tumorigenesis assays. The tumor weight was assessed following the sacrifice of the mice. With the use of a digital caliper, the tumor volume (per group) was determined and computed as length × width^2^ × 0.5.

### 5.5 Lectin enrichment and blotting

Protein samples were extracted from HNSCC cell lines via IP lysis buffer and then incubated with various biotinylated lectins (LCA, Con A, and PHA‐L, 10 µg) (Vector Laboratories, Inc.) for 24 h at 4°C. This solution was then mixed with 50 µL of streptavidin magnetic beads (Pierce, Thermo Fisher Scientific) for 1 h at room temperature. After antigen recovery, the supernatant containing biotinylated lectins and targeted proteins was next submitted to immunoblot analysis. After that, the transferred membrane was treated with primary antibodies (such as PD‐L1 or biotinylated lectins) and then incubated with horseradish peroxidase streptavidin as a secondary antibody.

### 5.6 Cell fractionation

For extraction of cytoplasmic, membrane, and nuclear proteins, HNSCC cells cultured in 10‐cm dishes were collected and spun down at 350 × *g* for 5 min, after which the cell pellet was resuspended in phosphate‐buffered saline (PBS). (1 × 500 µL). Protein was extracted from this suspension of cells by using a cell fractionation kit according to the manufacturer's instructions.

### 5.7 Tumor‐infiltrating lymphocyte (TIL) assay

To analyze CD4^+^ and CD8^+^ T‐cell infiltration in the tumor microenvironment, immunocompetent C3H mice were implanted with the mouse HNSCC cell line SCC7 (2 × 10^6^) transfected with vector, WT, or mutant plasmids in the right flank region for observation of tumor growth. Once the mice were sacrificed, the tumor weight was recorded. The tumor volumes were recorded every 4 days and calculated via the following formula: 0.5 × length (mm) × width (mm)^2^. In the TIL analysis, tumors from various groups were diced into small fragments, incubated with a tumor dissociation kit (Miltenyi Biotec) for 30 min, and subsequently filtered through a 70‐µm mesh to create a single‐cell suspension. For cell‐surface marker staining, the cell suspensions were washed twice with PBS and then incubated with fluorescent‐labeled antibodies (CD4, CD8, and Ki‐67) for 30 min on ice. For intracellular marker staining, the resuspended cells were fixed and permeabilized via a Cytofix/CytoPerm buffer kit (Cat# 554714, BD Bioscience). All analyses were conducted via flow cytometry (BD FACSCalibur), and the data were further analyzed via FlowJo software (FlowJo Vx.0.7).

### 5.8 Tumor cell‐mediated T‐cell exhaustion assay

By using the EasySep Human CD8^+^ T‐Cell Isolation Kit (STEMCELLTM Technologies), the isolation of CD8^+^ T cells from human peripheral blood samples was carried out. ImmunoCult‐XF T‐Cell Expansion Medium and human recombinant IL‐2 (STEMCELL Technologies) were selected for in vitro culture and activation of isolated CD8^+^ T cells, respectively. Next, flow cytometry was performed on these enlarged CD8^+^ T cells with anti‐CD3 and anti‐CD8 antibodies. For the functional experiments, CD8^+^ T cells were coincubated with HN6 cells at a ratio of 1:1 in 12‐well plates for 24 h. CD8^+^ T cells were then subjected to flow cytometry for exhaustion analysis by using anti‐LAG3 and anti‐TIGIT antibodies (Abcam), and the exhaustion score of the CD8^+^ T cells was finally evaluated through detection of the fluorescence intensity of each sample.

### 5.9 Statistical analysis

The Wilcoxon test, Kolmogorov‒Smirnov test, and Fisher's exact test were performed via the R platform (v.3.1.2). All experiments were repeated a minimum of three times to determine the reproducibility of the results. All error bars represent the SEM. Statistical analysis was performed via the Student's *t* test. A *p* value < 0.05 was considered statistically significant.

## AUTHOR CONTRIBUTIONS

Zhonglong Liu and Yue He designed the research, Zhonglong Liu and Xiaoyan Meng conducted the experiments, and Xiao Tang assisted in animal experiments and statistical analysis. Jian Zhang provided methodological supports (WES+AlloDriver). Zhiyuan Zhang and Yue He supervised the research together. All authors have read and approved the final manuscript.

## CONFLICT OF INTEREST STATEMENT

The authors declare no conflicts of interest.

## ETHICS STATEMENT

All procedures carried out in the current study involving human samples were in accordance with the principles of the Declaration of Helsinki, and written informed consent was obtained from all participants. Approval was granted by the Ethics Committee of Shanghai Ninth People's Hospital Affiliated to Shanghai Jiao Tong University School of Medicine with the number of “SH9H‐2021‐T384‐1” for human and “SH9H‐2022‐A900‐SB” for animal experiments, respectively.

## Supporting information



Supporting Information

## Data Availability

The raw data of WES generated in this study were deposited in Genome Sequence Archive (GSA) with accession ID HRA007967 and the raw data of bulk RNA‐seq of human HNSCC cell lines were deposited in GSA with accession ID HRA008941. Since these data are related to human genetic resources, raw data can be obtained directly by requesting and following the GSA guidelines for academic use at https://ngdc.cncb.ac.cn/gsa‐human/browse/HRA007967 and https://ngdc.cncb.ac.cn/gsa‐human/browse/HRA008941 after the user log in to the GSA database with the email address of the academic institution. The request will be responded within 2 weeks. Once access is granted, users have 6 months to download the data. The guidance for making a data access request of GSA for humans can be downloaded from https://ngdc.cncb.ac.cn/gsa‐human/document/GSA‐Human_Request_Guide_for_Users_us.pdf. Other relevant data are within the manuscript and its additional files, and are available from the corresponding author upon reasonable request.
