# Supplementary material for: A novel allosteric driver mutation of β‐glucuronidase promotes head and neck squamous cell carcinoma progression through STT3B‐mediated PD‐L1 N‐glycosylation
Source: MedComm (2020). 2025 Jan 19;6(2):e70062. doi: 10.1002/mco2.70062 (PMC11742429; doi:10.1002/mco2.70062)
Supplement: Supplementary file 1 — Supporting Information [file MCO2-6-e70062-s001.docx]

***A novel allosteric driver mutation of β-glucuronidase promotes head and neck squamous cell carcinoma progression through STT3B-mediated PD-L1 N-glycosylation***

Zhonglong Liu^# 1^, Xiaoyan Meng^# 1^, Xiao Tang^1^, Jian Zhang^2^, Zhiyuan Zhang^1^, Yue He^*1^

1 Department of Oral Maxillofacial & Head and Neck Oncology, Shanghai Ninth People’s Hospital Affiliated to Shanghai Jiao Tong University School of Medicine, Shanghai, China, 200011; National Clinical Research Center for Oral Disease, National Center of Stomatology

2 Medicinal Bioinformatics Center, Shanghai Jiao Tong University, School of Medicine, Shanghai 200025, China

Zhonglong Liu and Xiaoyan Meng contributed equally to this work. Namely, Zhonglong Liu and Xiaoyan Meng have equal contribution to this investigation

**Authors’ information**

**Zhonglong Liu**, DDS, Ph.D. Department of Oral Maxillofacial & Head and Neck Oncology, Shanghai Ninth People’s Hospital Affiliated to Shanghai Jiao Tong University School of Medicine, Shanghai, China, 200011. National Center of Stomatology, National Clinical Research Center for Oral Disease, National Center of Stomatology.

**Xiaoyan Meng**, DDS; Department of Oral Maxillofacial & Head and Neck Oncology, Shanghai Ninth People’s Hospital Affiliated to Shanghai Jiao Tong University School of Medicine, Shanghai, China, 200011. National Center of Stomatology, National Clinical Research Center for Oral Disease, National Center of Stomatology.

**Xiao Tang**, DDS; Department of Oral Maxillofacial & Head and Neck Oncology, Shanghai Ninth People’s Hospital Affiliated to Shanghai Jiao Tong University School of Medicine, Shanghai, China, 200011. National Center of Stomatology, National Clinical Research Center for Oral Disease, National Center of Stomatology.

**Jian Zhang**, Ph.D. Medicinal Bioinformatics Center, Shanghai Jiao Tong University, School of Medicine, Shanghai 200025, China

**Zhiyuan Zhang**, DDS, MD, Ph.D. Department of Oral Maxillofacial & Head and Neck Oncology, Shanghai Ninth People’s Hospital Affiliated to Shanghai Jiao Tong University School of Medicine, Shanghai, China, 200011. National Center of Stomatology, National Clinical Research Center for Oral Disease, National Center of Stomatology.

**Yue He,** DDS, MD, Ph.D. Department of Oral Maxillofacial & Head and Neck Oncology, Shanghai Ninth People’s Hospital Affiliated to Shanghai Jiao Tong University School of Medicine, Shanghai, China, 200011. National Center of Stomatology, National Clinical Research Center for Oral Disease, National Center of Stomatology.

**Correspondence to Prof. Yue He DDS, MD, PhD;**

Department of Oral Maxillofacial & Head and Neck Oncology, Shanghai Ninth People’s Hospital Affiliated to Shanghai Jiao Tong University School of Medicine, Shanghai, National Center of Stomatology, National Clinical Research Center for Oral Disease, National Center of Stomatology, China, 200011.

Tel: +86(0)21 23271699×5656

Email: william5218@126.com

**Supplementary figures**

**Figure S1**. A-B. Presentation of the quality score across all bases acquired from whole-exome sequencing (WES) of the HNSCC tumor and paired normal specimens. C. Quality control of the exome region, including exome size, coverage/average depth, mapped data, and target capture specificity.

**Figure S2**. Verification of GUSB-H351Q mediated growth-promoting effect in HN30 cells by using CCK8. (***, p≤0.001)

**Figure S3.** The influence of H351Q mutation on the GUSB protein stability and turnover rate in the presence of cycloheximide (CHX) at designed timepoints.

**Figure S4**. A. Protein misfolding and ER stress analysis by using western blot through detection of ER homeostasis marker (Bip, HSP40, HSP90). B. ERAD analysis of GUSB-WT and GUSB-H351Q transfected cells by using western blot.


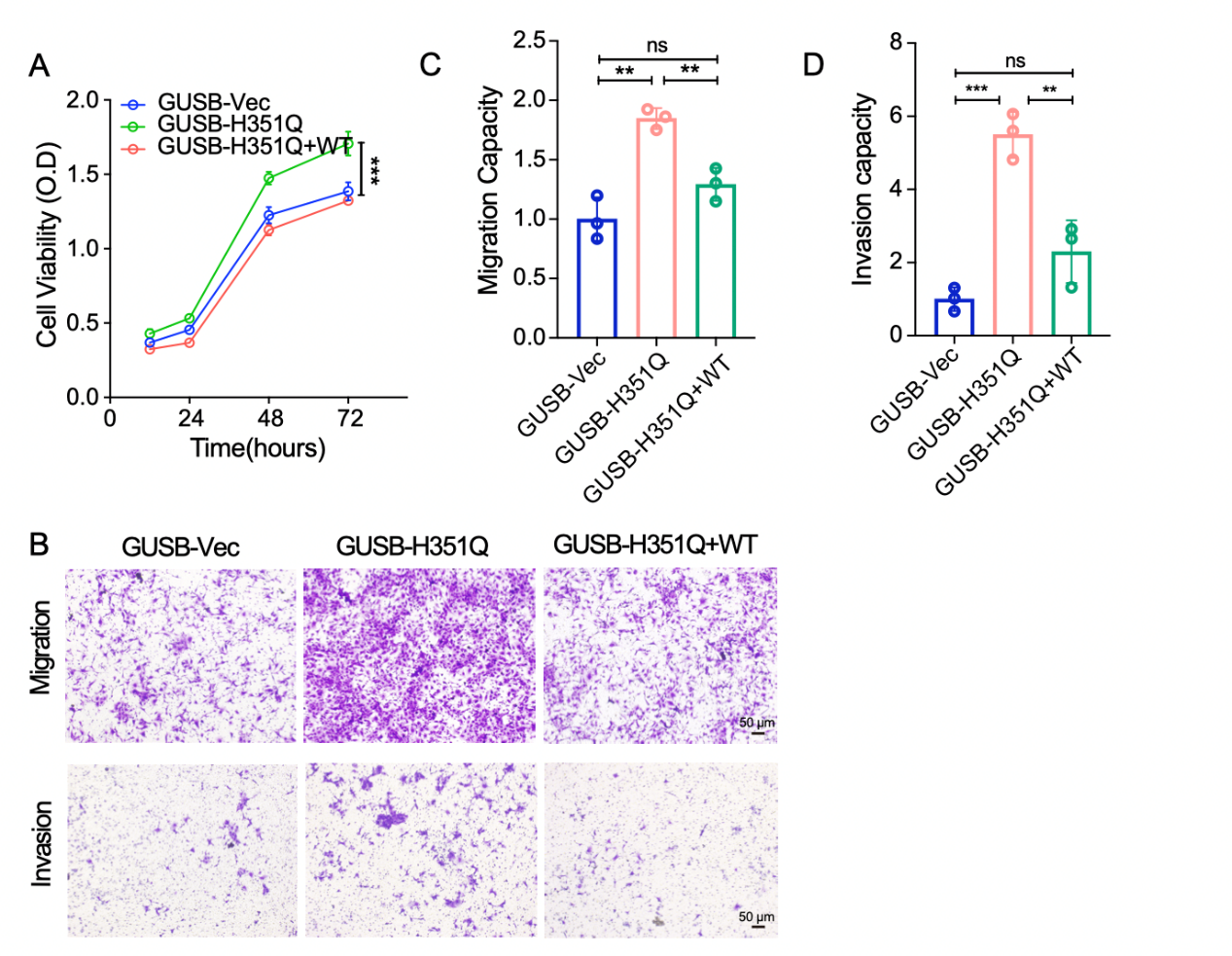


**Figure S5**. Rescue experiments of GUSB-WT on H351Q mediated malignant phenotype of tumor cells. A. CCK8 analysis of HN6 cells transfected with GUSB Vector, H351Q, H351Q+WT at different timepoints. B. Migration and invasion analysis of HN6 cells with different plasmids transfection at 24 and 12 h, respectively. Scale bar, 50μm. C-D. Semiquantitative analysis of the cell migration and invasion. (**, p≤0.01; ***, p≤0.001)


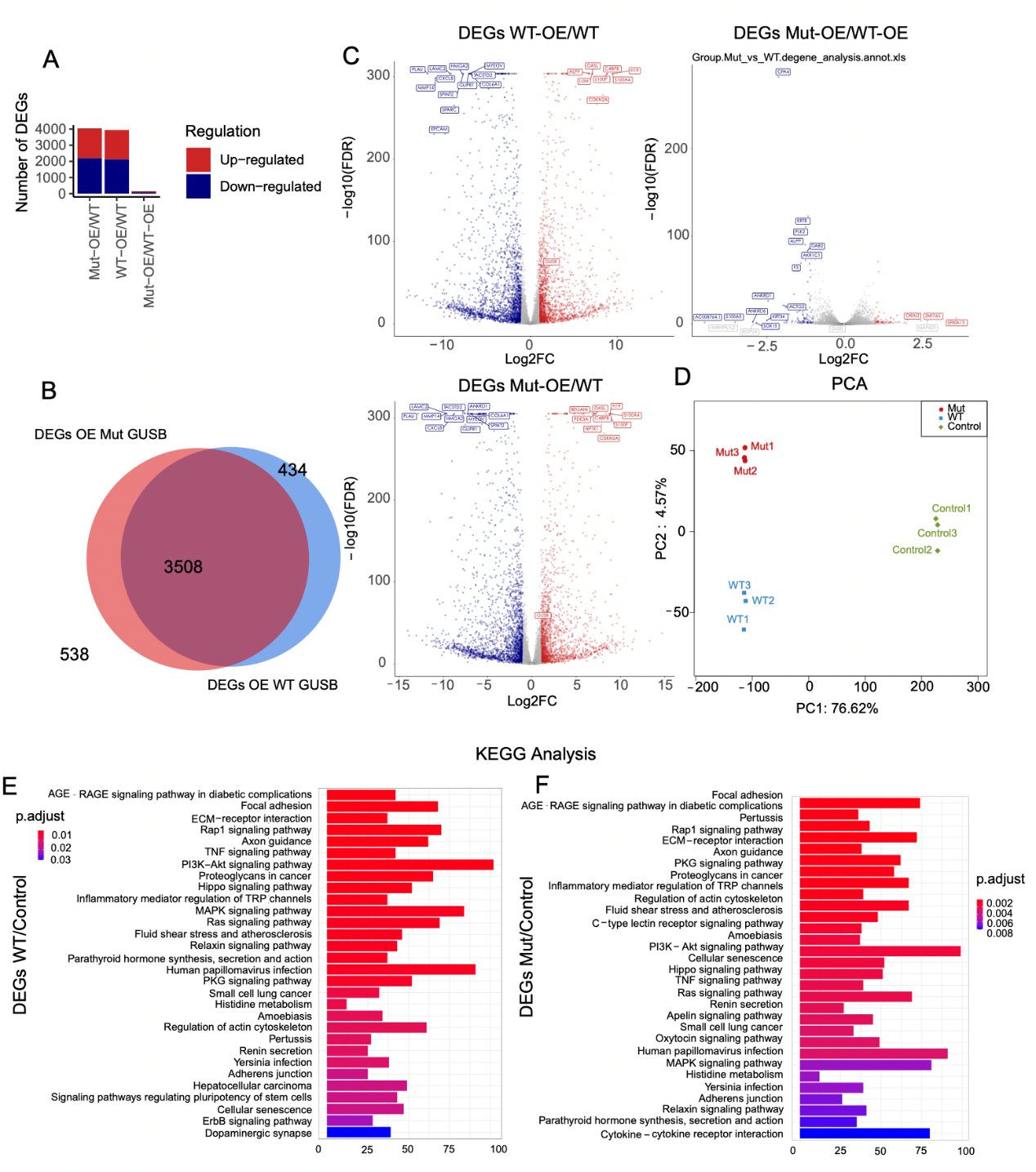


**Figure S6**. RNA sequence analysis of HN6 cells transfected with Vector, WT and H351Q. A-B. Number of differentially expressed genes (DEGs) among three groups. C. Presentation of DEGs in volcano plots. D. PCA analysis of the data in various groups. E. KEGG pathway enrichment analyses.

**Figure S7**. A. Verification of the GUSB-H351Q mediated protein overexpression of STT3B in HN30 and Cal-27 HNSCC cell lines. B. Transcription analysis of STT3B in HN6 cells transfected with GUSB-Vector, H351Q, H351Q+WT (**, p≤0.05). C. Western blot detection of STT3B in HN6 cells with different transfections. D. Protein degradation analysis of STT3B in GUSB-WT and GUSB-H351Q transfected cells pretreated with protein synthesis inhibitor (Cycloheximide, CHX), and then incubated with UPS inhibitor (MG132), ALP inhibitor (hydroxychloroquine, HCQ) and ERAD inhibitor (Bortezomib, BTZ).

**Figure S8**. Co-IP experiments of protein samples acquired from HN6 cells transfected with GUSB-Vector, H351Q, H351Q+WT.

**Figure S9**. Cell fractionation and Co-IP analysis to uncover the GUSB-H351Q dominated PD-L1 glycosylation in different cellular locations (nucleus, cytoplasm and cytomembrane).

**Figure S10**. H-Score of STT3B in immunohistochemistry analysis among low, intermediate and high groups. (****, *p*≤0.0001)

**Figure S11**. Differential metabolites between the GUSB-WT and H351Q tumors are presented as heatmaps of hierarchical clustering analysis.

**Figure S12**. Correlation-heatmap showed the intrinsic relations among various metabolites.


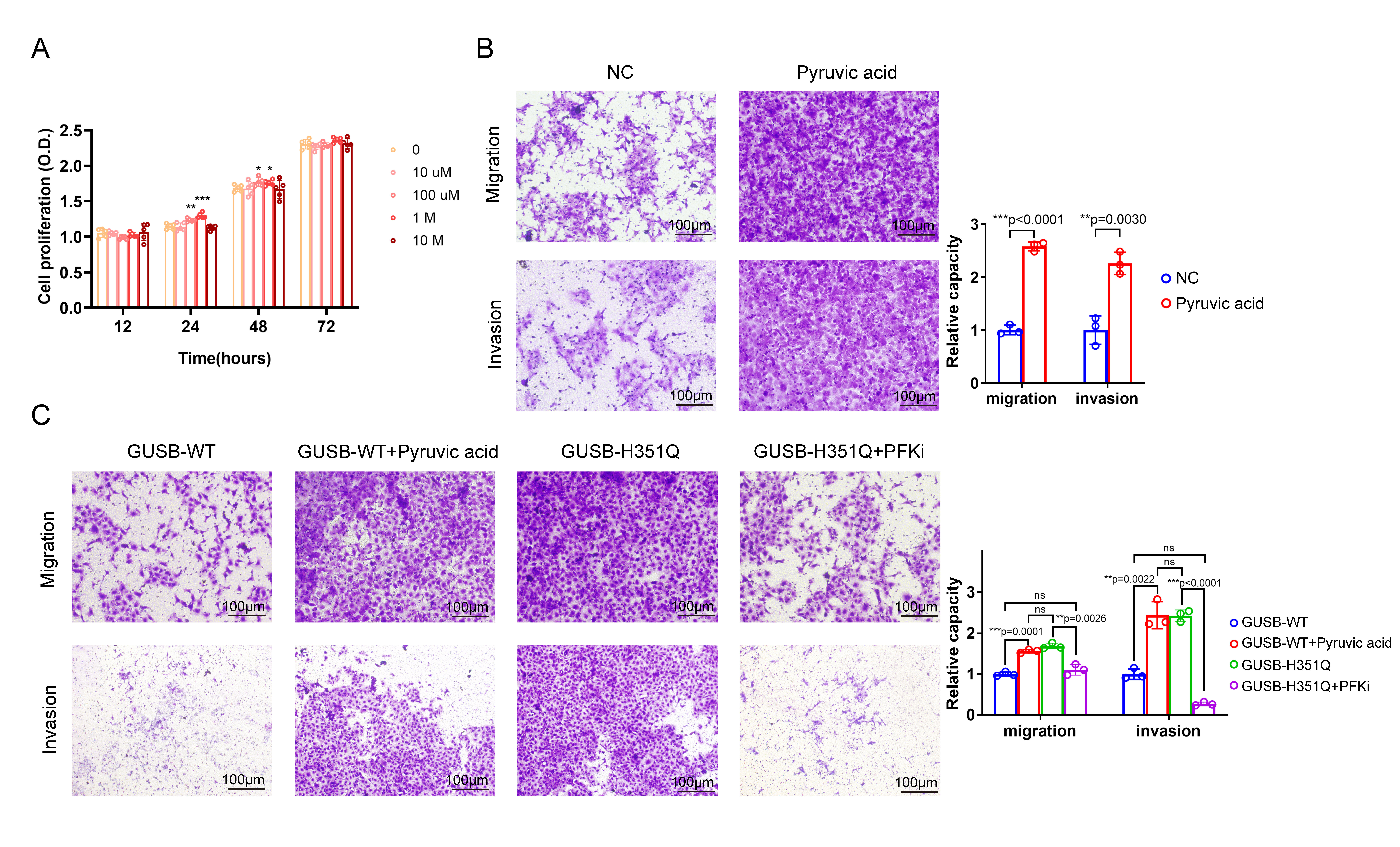


**Figure S13**. A. CCK8 analysis of HN6 cells treated with pyruvic acid at different concentrations at different timepoints. B. Migration and invasion analysis of HN6 cells with or without pyruvic acid treatment at 24 h. Scale bar, 100 μm. The quantitative analysis of cell migration and invasion capabilities are showed on the right. C. Migration and invasion analysis of HN6 cells with indicated treatment at 24 h. Scale bar, 100 μm. The quantitative analysis of cell migration and invasion capabilities are showed on the right. (*, p ≤ 0.05; **, p ≤ 0.01; ***, p ≤ 0.001)

**Supplementary table**

**Supplementary table 1. Patient characteristics.**

| Patients | Gender | Age | pTNM | Primary site | Pathological  diagnosis | HPV Statue | Tobacco  Statue | Alcohol Status |
| --- | --- | --- | --- | --- | --- | --- | --- | --- |
| 1 | Male | 49 | T1N0M0 | Left tongue | SCC | - | Yes | Yes |
| 2 | Male | 33 | T2N1M0 | Left tongue | SCC | - | No | No |
| 3 | Male | 67 | T2N3M0 | Right tongue | SCC | - | Yes | No |
| 4 | Female | 40 | T2N0M0 | Right tongue | SCC | - | No | No |
| 5 | Female | 61 | T1N1M0 | Left tongue | SCC | - | No | No |
| 6 | Male | 74 | T2N0M0 | Left tongue | SCC | - | Yes | Yes |
| 7 | Male | 56 | T3N1M0 | Right tongue | SCC | - | No | Yes |
| 8 | Female | 47 | T2N3M0 | Right tongue | SCC | - | No | No |
| 9 | Female | 68 | T1N2M0 | Left tongue | SCC | - | No | No |
| 10 | Male | 70 | T2N1M0 | Left tongue | SCC | - | Yes | Yes |

SCC: squamous cell carcinoma

**Supplementary methods**

**1. Cell culture**

The human HNSCC cell lines HN6 and Cal-27, the mouse HNSCC cell line SCC7 and the human embryonic kidney 293T cell line were obtained from the American Type Culture Collection (ATCC). HN6, Cal-27, SCC7 and 293T cells were cultured in Dulbecco’s modified Eagle’s medium (DMEM) supplemented with 10% fetal bovine serum (FBS) and 1% penicillin and streptomycin at 37 °C in a humidified atmosphere containing 5% CO_2_.

**2. Plasmid construction and transfection**

The construct pcDNA3.1(+) was purchased from TaKaRa Technology. The mutant cDNAs were acquired by using the QuikChange Site-Directed Mutagenesis Kit. Subsequently, the wild type and mutant cDNAs were effectively amplified by using 2 x Pfu (PCR) Master Mix. Then, we subcloned amplified cDNAs into the pCDH-CMV-MCSEF1-copGFP vector at the XbaI and BamHI sites (System Biosciences). All plasmids used in current study were verified through sequencing. For establishment of stable cell line, HNSCC cells were infected with viral particles and polybrene (10 μg/ml), and then undergone the selection of positive stable clones by puromycin and further single-cell dilution cloning.

**3. Transmission electron microscopy**

HN6 cells subjected to different stimuli were scraped and then centrifuged for 10 min at 3000 rpm. The cell pellet was fixed in 2.5% glutaraldehyde (Solarbio Life Sciences, China) for 2 h, followed by dehydration with a graded ethanol series and embedding in Epon618. Ultrathin sections (70−100 nm) of the collected cells were stained with lead citrate and uranyl acetate and then visualized via transmission electron microscopy (TEM; JEOL-1230; JEOL).

**4. Lentivirus production and transfection**

In dishes with a diameter of 10 cm, we transfected 293T cells with the human wild-type or mutant GUSB expression vectors in combination with lentiviral packaging vectors containing psPAX2 and pMD2.G by using X-tremeGENETM 9 DNA Transfection Reagent. Following the transfection for 4-6 hours, the culture supernatant of modified 293T cells was substituted with fresh medium. Following an incubation period of 48-72 hours, the supernatants of cells transfected with viruses were collected and filtered through a 0.45 mm syringe filter. Subsequently, the viruses were immediately used to infect HN6 cells or stored at -80 °C. If necessary, the viruses were concentrated through ultracentrifugation with a rotational speed of 28,000 rpm for 2 hours at a temperature of 4 °C. The pellets were resuspended in PBS supplemented with 2% FBS and divided into smaller aliquots for storage at -80 °C. The HN6 cells were cultured in 6 cm diameter dishes and infected with the concentrated lentivirus on the following day. To enhance the infection efficiency, polybrene was introduced at a concentration of 8 mg/mL to the infected cells. To achieve enhanced infection efficiencies, flow cytometry sorting (Beckman) was used to sort the infected HN6 cells. Ultimately, a GFP-positive rate exceeding 95% was observed in these stable HN6 cell lines.

**5. Cell proliferation analysis**

In 96-well plates, cells were seeded at a density of 4,000 cells and incubated for 24 hours. The specified compound or the vehicle control was subsequently employed to treat the cells and incubated for an additional 72 hours. The CellTiter 96 Aqueous One Solution Cell Proliferation Assay (MTS) (Promega) was used to assess the inhibition of cellular proliferation induced by the treatments. To validate the effect of the mutant on HNSCC cell growth, we cultured stable HN6 cells and corresponding control cells at a density of 600 cells in 96-well plates containing medium with 1% FBS to minimize serum interference. Then, the CellTiter 96 Aqueous One Solution Cell Proliferation Assay (MTS) was employed to detect cell growth at the specified time points. The assays were performed in accordance with the manufacturer's instructions, and the absorbance value (optical density) of each well was measured at 490 nm using a microplate reader. The baseline value was obtained by subtracting the absorbance at 630 nm from this measurement. All experiments were conducted at least three times.

**6. CCK8 analysis**

The cells were cultured in 96-well plates at a density of 1000 cells per well. For measurement, the culture medium was removed, and 110 μL of the novel culture medium was incubated with a CCK8 kit at a ratio of 10:1 for 2 h at 37 °C. The OD values of the treated samples were subsequently measured with a microplate reader at 450 nm. The difference in cell viability was analyzed by Student’s t test.

**7. Western blotting and immunoprecipitation**

Cells were lysed using a 2x SDS lysis buffer containing 100 mM Tris HCl (pH 6.8), 200 mM DTT, 4% SDS, 0.2% bromophenol blue, and 20% glycerin. The proteins in the samples underwent separation using sodium dodecyl sulfate-polyacrylamide gel electrophoresis (SDS-PAGE) and were subsequently transferred onto PVDF membranes (Millipore) through electroblotting. We blocked PVDF membranes with 5% non-fat milk at room temperature for 1 hour, followed by incubation with the appropriate primary antibody (*anti-Flag, 1:1000, Biorbyt; anti-GAPDH, 1:1000, CST; anti-STT3B, 1:1000, Proteintech; anti-α-Tubulin, 1:1000, CST; anti-GUSB, 1:1000, Abcam; anti-PD-L1, 1:1000, Abcam; anti-Na/K ATPase, 1:1000, CST; anti-Lamin A, 1:1000, CST*) at 4 °C overnight. After conducting additional TBST washes, we incubated membranes with the corresponding horseradish peroxidase-conjugated secondary antibodies for 1 hour at room temperature.

For co-IP experiments, cells were lysed using IP Lysis Buffer (Thermo Fisher Scientiﬁc) and protease inhibitor. Immunoprecipitation was performed by incubating the protein suspension (200 μl) with anti-Flag Dynabeads (25 μl) (Bimake.cn, China) overnight at 4 °C. Acquired samples were subsequently subjected to immunoblotting with anti-STT3B (1:1000, Proteintech), anti-Flag (1:1000, Biorbyt) and anti-α-Tubulin (1:1000, CST). Scanning of PVDF membranes applied an Odyssey infrared imaging platform (LI-COR Biosciences, Lincoln, NE, USA). The protein bands were evaluated and semi-quantified by using the ImageJ software.

**8. In vitro immunofluorescence analysis**

HNSCC cells were cultured in confocal dishes (NEST, China) and then transfected with vector, wild-type or H351Q mutation plasmids when the cell confluence reached 60–70%. These genetically modified cells were fixed with ice-cold methanol or 4% paraformaldehyde for 15 min, permeabilized for 10 min with 0.1% Triton X-100, and finally blocked for 30 min with 1% BSA. The cell samples were incubated with primary antibodies, such as those against GUSB, STT3B, calreticulin, GM130, LAMP1, PD-L1 and Con A, overnight at 4 °C. After three washes with PBS, the cells were stained with secondary antibodies conjugated with Alexa Fluor 488- or 647-conjugated (1:500, Jackson Lab) or streptavidin, DyLight™ 488 (10 µg/ml, Vector Lab), for 1 h at RT, followed by counterstaining with DAPI for 10 min. All images were acquired under a confocal laser scanning microscope (CLSM, Leica SP8).

**9. Colony formation assay**

For the colony formation experiments, HN6 cells at a density of 1×10^3^ cells/mL were seeded into 6-well plates and cultured for 2 weeks at 37 °C with 5% CO_2_. The colonies were fixed with ice-cold methanol for 10 min, washed twice with PBS, and stained with 0.1% crystal violet for 15 min. Colonies with ≥50 cells were counted and captured under a microscope.

**10. Cell migration assay**

HNSCC cells with different genetic modifications were seeded in Transwell chambers with medium containing 0% FBS, and these chambers were then cultured with 10% FBS as a chemoattractant in 24-well plates. After 24 h, the invading cells at the bottom of the chamber were fixed with 4% paraformaldehyde for 15 min, washed with PBS three times, and finally stained with 0.1% crystal violet at RT for 1 h. The number and ratio of migrated cells were calculated in 5 randomly selected fields via microscopy.

**11. Wound healing assays**

For the wound healing assay, genetically modified cells were seeded in 6-well plates for 24 h. The adherent cells were scratched and photo-recorded. Then, the cells were returned to the cell incubator. After 24 h and 48 h, the cells were washed twice, and five fields were randomly selected under ×40 microscope for photo-recording.

**12. RNA-Seq analysis**

Whole RNA was extracted from the GUSB-modified cell samples via a RNeasy Plus Mini Kit (Qiagen) following the manufacturer’s instructions. The acquired RNA was then subjected to quality control and subjected to RNA-Seq analysis on an Illumina system by Origingene (Shanghai, China). The RNA was subsequently reverse transcribed for cDNA library construction and further sequencing. All the raw sequencing reads were first filtered to acquire the clean reads and then stored in the FASTQ format.^1^ Bowtie2 and HISAT were applied to map the clean reads to the corresponding genes and genomes, respectively.^2^ RNA-Seq by expectation-maximization (RSEM) was selected to evaluate the expression level of genes in terms of the fragments per kilobase per million (FPKM) values.^3^ Subsequently, the differentially expressed genes (DEGs) among the various groups were screened via the NOI-Seq method under the following conditions: fold change ≥ 2 and probability divergence ≥ 0.8. Gene Ontology (GO) analysis was performed using the Gene Ontology database, and pathway enrichment analysis was carried out on the KEGG pathway database.

**13. Proteomics study and bioinformatics analysis**

The cell precipitates were treated with a mixture containing urea (8 M), Tris-HCl (100 mM, pH 8.5), and protein inhibitors to achieve protein lysis and denaturation, and then subjected to ultrasonic oscillation on ice to disrupt the interactions between proteins or between DNA and proteins. The protein mixture then underwent reduction and alkylation through reaction with 5 mM Tris (2-​carboxyethyl)​ phosphine hydrochloride (TCEP) and 10 mM iodoacetamide at RT for 30 minutes. The peptide mixtures were then digested with trypsin, desalted with MonoSpin™ C18, and finally dissolved in 100 mM HEPES buffer at a pH of 8.0.

A quantitative colorimetric peptide assay was used to measure the peptide concentrations. A TMT6plex amino reactive kit was used to label the peptide mixture according to the manufacturer’s instructions. The complex peptide mixture was separated into 8−10 fractions. Each fraction was then subjected to mass spectrometry analysis via a pulled-tip analytical column and an Easy-nLC 1200 nano-HPLC system at a preset temperature of 55 °C and a predetermined program with an Orbitrap Eclipse Tribrid mass spectrometer according to the manufacturer’s instructions.

The acquired MS/MS data were calculated on the basis of the UniProt Knowledgebase by using MaxQuant (V1.6.10.43). The R4.0.0 platform was selected for the annotation of the proteomic data from MaxQuant. Specifically, empirical Bayes moderated t tests were performed to calculate the P value of the data, and the Benjamini and Hochberg platforms were used to adjust the P value via the limma package. A P value ≤ 0.05 was considered to indicate differential expression. Gene Ontology (GO) analysis was carried out via the DAVID platform.

**14. qRT–PCR assays**

An Axygen Total RNA Isolation Kit was used to extract the total RNA from HNSCC cell lines, and a PrimeScript™ RT Reagent Kit was used for further reverse transcription to synthesize the cDNA. Quantitative reverse transcriptase PCR (qRT–PCR) experiments were conducted via the One Step SYBR@ PrimeScript@ PCR Kit on the Applied Biosystems StepOne^TM^ platform (Invitrogen, USA). Differential expression of the STT3B gene (primer sequences: forward “TGCGACTGATGTTGACTTTGACTCC” and reverse “TCCTCATCACTGCTGTCCTCCAC”) was evaluated via the 2^−ΔΔCT^ formula, which was normalized to the expression level of the housekeeping gene GAPDH (Sangon Biotech, Shanghai, China).

**15. Immunohistochemistry (IHC) and immunofluorescence (IF)**

Pathology slides were deparaffinized, and antigen recovery was performed by using Tris-borate-EDTA, followed by blocking of nonspecific protein interactions with 10% goat serum at RT for 30 min. The slides were then treated with anti-STT3B (1:250, Proteintech) overnight at 4 °C, followed by incubation with a secondary antibody for 1 h at RT. After being washed with distilled water, the sections were stained with hematoxylin. For IF analysis, the slides were sequentially stained with anti-PD-L1 (1:100, Abcam), Alexa Fluor 488-conjugated secondary antibody (1:400, Jackson Lab), rhodamine-conjugated Con A antibody (5 mg/ml, Vector Lab) and DAPI (10 µg/ml, Sigma‒Aldrich), and finally, images were captured via CLSM (Leica SP8).

The semiquantitative estimation of the IHC- and IF-stained sections was carried out by a pathologist in a blinded way according to calculation methods for the ratio of positive cells in five randomly selected fields. The results were analyzed via the histochemistry score (H-Score) as follows: (percentage of cells of weak intensity X 1) + (percentage of cells of moderate intensity X 2) + (percentage of cells of strong intensity X 3). For the IF analysis, we calculated the positive ratio of Con A^+^/PD-L1^+^ cells to assess the extent of PD-L1 glycosylation.

**16. Metabolomic analysis by LC‒MS/MS**

To uncover the metabolomic heterogeneity between GUSB-WT and H351Q tumors, we performed steady-state untargeted metabolomics analysis. Tumors resected from C3H mice were washed with ice-cold PBS three times and then cut into pieces for metabolite extraction. All samples were extracted with a 100 μL aliquot, 300 μL of methanol and 10 μL of the internal standard (3.0 mg/mL, DL-o-chlorophenylalanine). The samples were then centrifuged at 15,000 rpm for 10 min at 4 °C to collect the supernatant for further liquid chromatography‒tandem mass spectrometry (LC‒MS/MS) analysis, which was performed via a Vanquish UHPLC system (Thermo Fisher) incorporating an Orbitrap Q Exactive instrument (Thermo Fisher) in both positive and negative ion modes.

The raw data generated by UHPLC‒MS/MS were analyzed via Compound Discoverer 3.1 (CD3.1, Thermo Fisher) through peak alignment/picking and metabolite quantification. The online Human Metabolome Database (https://hmdb.ca) and KEGG database (https://www.genome.jp/kegg/) were used to identify the metabolites by aligning the molecular mass data. PCA, PLS-DA, and loading plots were generated via SIMCA-P software (version 13.0; Umetrics, Umea, Sweden). Differentially abundant metabolites were screened according to the VIP values acquired from the PLS-DA model and statistical analysis (VIP score of 1 and P value of 0.05).

**References:**

1. Cock PJ, Fields CJ, Goto N, et al. The Sanger FASTQ file format for sequences with quality scores, and the Solexa/Illumina FASTQ variants. *Nucleic Acids Res*. 2010;38(6):1767-1771.

2. Langmead B, Salzberg SL. Fast gapped-read alignment with Bowtie 2. *Nature Methods*. 2012;9(4):357-359.

3. Li B, Dewey CN. RSEM: accurate transcript quantification from RNA-Seq data with or without a reference genome. *BMC bioinformatics*. 2011;12:323-339.
